# Case.—Amputation at the Shoulder Joint.—Recovery

**Published:** 1869-03-01

**Authors:** Hiram Wanzer

**Affiliations:** 172 West Twelfth Street; Chicago, Ills.


### ORIGINAL COMMUNICATIONS.

### *Case.—Amputation at the Shoulder-joint.—Recovery.*

BY HIRAM WANZER, M. D., CHICAGO, ILLS.

I was called, Dec. 8th ult. to Mr. C. Jerman, aged 51. He was rather spare, small in stature, with constitution not vigorous. He had suffered from the age of 16, with discharging ulcers upon one leg and foot, which had healed two years since. He was one of the employés of Messrs. Moody & Church. While engaged at a surfacing planer, which has a cylinder with three cutting knives, performing some 3,500 revolutions per minute, the fingers of the right hand were caught in the machinery, and its deadly work was not finished until the hand, forearm, and arm, were drawn in and severed by piecemeal, as far as the upper fourth of the humerus. Further advance and destruction were prevented by the contact of the chest and shoulder against the planer.

The patient was taken to a druggist, who administered morphia and diffusible stimulants. There was now sufficient pulse and vitality to justify operative procedure, but the surroundings being unfitted, he was taken to his residence, one and a half miles. The length of time, and the degree of suffering, during his transit home, were succeeded by shock, and such extreme prostration of the vital powers, that operative measures were thought contra-indicated.

Drs. H. T. and E. W. Lee, were called in consultation. I improved the moments, before their arrival, in the administration of strong coffee, and diffusible stimulants, which had but little effect, either in increasing the heart's action, or in arousing the vital energies. I thought it judicious to follow the opinions of some of our most eminent surgical authorities, in waiting for warmth of the surface, color, pulse at the wrist, consciousness, and a greater degree of reaction, especially before proceeding with such a formidable operation. It was the opinion of the counsel, that no further reaction could be obtained—that operative measures were only indicated. Drs. Lee administered chloroform to anesthesia. During its early inhalation the almost imperceptible, fluttering pulse, became increased in tone and volume. There had been very little hæmorrhage in consequence of the torsion of the vessels. The brachial artery was pulsating at the bottom of the wound; the torn terminal branches of the brachial plexus were lying by its side. The question arose, whether to disarticulate the head and remaing shaft of the humerus by incision, or separate the soft parts from their circumference. I chose the latter to avoid opening the cavity of the joint, and prevent so large a suppurating surface. After ligating the brachial artery, the soft parts were carefully detached from the bone by the fingers and scalpel. There were difficulties encountered in reaching and perforating the capsular ligament in consequence of its distance and density. At this juncture we were suddenly stopped. The pulse forsook the wrist, respiration momentarily suspended; showing the well marked toxic action of the chloroform upon the blood, nerve centers and respiratory organs; free admission of atmospheric air, cold jets of water were thrown upon the surface, with recumbency. The patient soon gasped; the pulse was again perceptible at the wrist; respiration re-established, the operation was resumed and finished. Considering the lesion to the blood vessels, caused by the accident and operation, the hæmorrhage was insignificant, the brachial artery only required ligation. The flaps handsomely formed by the machinery, were coapted by several interrupted sutures, &c. Heat and derivatives were now applied to the cold surface and extremities. He speedily reacted from the shock of the injury and operation, when a cordial stimulant was given.

9th. 8 A. M. Visited patient with Dr. E. W. Lee, found the pulse 120 per minute, with surprising strength and volume; there was general warmth and moisture upon the surface. He had enjoyed some refreshing sleep. The following day there was an erysipelatous glow, with heat and tenderness in the axilla, which soon disappeared, by cold lotions and 20 gtt. every 3 hours, of Tinct. Ferri Muriatis, alternated with Rhine wine, his accustomed beverage, which were all the internal medicines given throughout the treatment. His appetite daily increased; the secretions normal. A small portion of the wound united by the first intention, with very little suppuration. I ascribe his speedy recovery much to his former uniform habits, the plasticity of the blood, and to our entire withholding of all narcotic remedies.

*Remarks.*—As stated, morphia was given by the apothecary. That would once have been my procedure, until surgical experiences taught me that narcotics, under the circumstances, not only allay *pain*, but they obtund nerve sensibility; they often paralyze the brain and heart; frequently they conduct our patient into a quiet sleep, which knows no waking. So seductive, *then*, is their action, it matters not how minute the dose, it is difficult to determine whether our patient is suffering from the pre-existing shock, or from the action of the medicine, so contra-indicated, as I believe them to be, for *pain*, after traumatic injuries. I have abandoned them for medicines that exalt the vital actions. *Pain* then becomes a therapeutic agent, a priceless stimulant to our patient, an excitor to organic action in the struggling reaction. Pain is a conflict for the mastery, that he often feels long after the pulse has left the wrist, and frequently abiding until the spirit takes its flight. I find, in searching the records of some of our surgical authorities, there are conflicting opinions about operating before reaction is established. I am confident my surgical career would have been brighter and more successful, had I used less opium and its alkaloids, and had I frequently acted with a greater degree of promptness, as in the case reported. In waiting too long for reaction, it often never came. In being too conservative, trying to save a worthless member, the patient has often sunk from pyemia, and the exhausting suppuration; or he has often passed, before this process was established, into traumatic delirium, tetanoid symptoms and death.

172 West Twelfth Street.

